# The Impact of Protozoan Predation on the Pathogenicity of *Vibrio cholerae*

**DOI:** 10.3389/fmicb.2020.00017

**Published:** 2020-01-21

**Authors:** Gustavo Espinoza-Vergara, M. Mozammel Hoque, Diane McDougald, Parisa Noorian

**Affiliations:** ^1^Faculty of Science, The ithree Institute, University of Technology Sydney, Sydney, NSW, Australia; ^2^Faculty of Science, Singapore Centre for Environmental Life Sciences Engineering, Nanyang Technological University, Singapore, Singapore

**Keywords:** protozoan predation, virulence, *Vibrio*, adaptation, pathogenicity, heterotrophic protist

## Abstract

In the aquatic environment, *Vibrio* spp. interact with many living organisms that can serve as a replication niche, including heterotrophic protists, or protozoa. Protozoa engulf bacteria and package them into phagosomes where the cells are exposed to low pH, antimicrobial peptides, reactive oxygen/nitrogen species, proteolytic enzymes, and low concentrations of essential metal ions such as iron. However, some bacteria can resist these digestive processes. For example, *Vibrio cholerae* and *Vibrio harveyi* can resist intracellular digestion. In order to survive intracellularly, bacteria have acquired and/or developed specific factors that help them to resist the unfavorable conditions encountered inside of the phagosomes. Many of these intra-phagosomal factors used to kill and digest bacteria are highly conserved between eukaryotic cells and thus are also expressed by the innate immune system in the gastrointestinal tract as the first line of defense against bacterial pathogens. Since pathogenic bacteria have been shown to be hypervirulent after they have passed through protozoa, the resistance to digestion by protist hosts in their natural environment plays a key role in enhancing the infectious potential of pathogenic *Vibrio* spp. This review will investigate the current knowledge in interactions of bacteria with protozoa and human host to better understand the mechanisms used by both protozoa and human hosts to kill bacteria and the bacterial response to them.

## Introduction

*Vibrio* spp. are metabolically versatile bacteria that inhabit the aquatic environment. They can be found in association with living organisms as well as with abiotic sediments and surfaces. *Vibrio* spp. have been associated with an array of organisms, including zooplankton and phytoplankton, crustaceans such as copepods, bivalves such as oysters and mussels, plants, fishes, and even water birds (Halpern et al., 2008; Senderovich et al., 2009; Vezzulli et al., 2010; Lutz et al., 2013). *Vibrio* spp. in their environment interact with heterotrophic protozoa, which are specialized eukaryotic cells that can be found in a wide variety of environments. Phagotrophic protozoa are competent grazers, consuming large numbers of prey, sometimes ingesting several times their own body weight (Vickerman, 1992).

Different environmental niches accommodate different predators and prey. For example, protozoa that are mainly surface-attached specialize on feeding on attached bacteria or biofilms, while suspension feeding protozoa consume planktonic bacteria, and some can feed on planktonic or biofilm cells (Finlay, 2001; Parry, 2004; Sigee, 2005). Some bacterivorous protozoa feed selectively on prey using a variety of different mechanisms (Caron et al., 1982; Sibille et al., 1998; Hahn and Höfle, 2001). For example, amoeba use specific protein receptors for recognition of bacterial prey (Pan et al., 2018) and internalize bacteria into phagosomes using actin microfilament-dependent engulfment (Clarke and Maddera, 2006). Other protists, such as ciliates, do not discriminate prey and package them into food vacuoles using ciliary motion (Gray et al., 2012). Flagellates draw their prey toward the base of the flagellum and into an oral groove by creating a strong current. Some filter-feeding flagellates use a collar of tentacles located at the base of the flagellum that allows the smallest prey particles to pass (Chrzanowski and Simek, 1990; Matz et al., 2002; Parry, 2004).

Once bacteria have been ingested, the expression of various factors can result in resistance to digestion and allows for intracellular growth, followed by escape to the extracellular environment. Many of the bacterial factors involved in digestion resistance and intracellular survival, growth, and escape from protozoa are also factors used by pathogenic bacteria during infection of other hosts (Cirillo et al., 1999; Al-Khodor et al., 2008; Adiba et al., 2010; Sun et al., 2018). This evidence supports the coincidental evolution hypothesis that states the virulence factors used by bacteria during *in vivo* infection are the result of adaptation to other ecological niches (Levin, 1996). Therefore, the study of the environmental factors that select for the emergence of virulence traits becomes relevant for better understanding of the emergence of pathogenic bacteria, including *Vibrio* spp.

In order to avoid protozoan predation, *Vibrio* spp. display various anti-grazing strategies, including the formation of biofilms (Matz et al., 2005; Sun et al., 2015), production of QS-regulated proteases such as PrtV (Vaitkevicius et al., 2006), secretion of ammonium and pyomelanin (Sun et al., 2015; Noorian et al., 2017), expression of the type VI virulence-associated secretion system (VAS) of *V. cholerae* first identified by Pukatzki et al. (2006) and the MARTX type III of *V. vulnificus* involved in the lysis of a wide range of eukaryotic cells, including amoebae (Lee et al., 2013).

Reports have shown that *V. cholerae* and *V. harveyi* can resist the intracellular environment in the amoeba, *Acanthamoeba castellanii* (Abd et al., 2004, 2005, 2007; Saeed et al., 2007; Shanan et al., 2016; Van der Henst et al., 2016, 2018) and the ciliate, *Cryptocaryon irritans* (Qiao et al., 2017) respectively. In addition, the release of *V. cholerae* in expelled food vacuoles (EFVs) has recently been demonstrated to increase fitness *in vitro* and *in vivo* (Espinoza-Vergara et al., 2019). The fact that the passage and release of pathogenic bacteria from the intracellular protozoan environment results in increased infectivity suggests that the exposure to intra-phagosomal factors may enhance virulence phenotypes.

Low pH, antimicrobial peptides (AMPs), and proteolytic enzymes, as well as reactive oxygen and nitrogen species (ROS/RNS) are examples of the factors encountered by *Vibrio* spp. when in the phagosome in predatory protozoa and during the innate immune defense in the gastrointestinal (GI) tract. Thus, the intracellular environment may serve as a pre-adaptive ecosystem for *Vibrio* spp. before entering a human host. This review will describe the similarities in the strategies used by protozoa and human hosts to kill bacteria and the molecular factors used by *Vibrio* spp. to overcome such stressors. The impact of exposure to the intra-protozoal environment on the infectivity of *V. cholerae* and other bacterial pathogens will also be discussed.

## Intracellular Survival of *Vibrio* spp.

Intracellular survival of *Vibrio* spp. has been demonstrated in various eukaryotic cells, including in the amoebae, *A. castellanii*, *Acanthamoeba polyphaga*, and *Naegleria gruberi* (Thom et al., 1992; Abd et al., 2007). *V. cholerae* O139 and O1 strains were shown to survive and grow within the cytoplasm of trophozoites and in cysts of *A. castellanii* (Thom et al., 1992; Abd et al., 2005, 2007). Furthermore, it has been shown that *V. cholerae* can access the contractile vacuole in *A. castellanii* and escape to the extracellular environment (Van der Henst et al., 2016). *V. harveyi* survives in the cytoplasm of the obligate parasitic marine ciliated protozoan, *C. irritans* (Qiao et al., 2017). *Vibrio splendidus* and *Vibrio parahaemolyticus* invade and survive intracellularly in other hosts such as oyster hemocytes (Duperthuy et al., 2011) and human epithelial cells (de Souza Santos and Orth, 2014), respectively.

Recent studies have investigated the intracellular mechanisms that mediate the survival and escape of *V. cholerae* from eukaryotic cells. Interestingly, virulence factors related to hemolytic activity and motility had a role in the intracellular survival of *V. cholerae* in *A. castellanii* (Van der Henst et al., 2018). In addition, OmpU, a major outer membrane protein that is needed for resistance to many stressors such as organic acids, bile, and AMPs as well as being a critical factor for the *in vivo* colonization of *V. cholerae* (Sperandio et al., 1995; Provenzano and Klose, 2000), plays a role in survival in protozoa. It was recently shown that OmpU is important for the expulsion of *V. cholerae* within food vacuoles of ciliate hosts, a fact that suggests that this protein might confer resistance to *V. cholerae* to the intra-phagosomal factors required for digestion (Espinoza-Vergara et al., 2019). Interestingly, it is also reported that OmpU is essential for *V. splendidus* host invasion and resistance to AMPs and is required for virulence in the oyster, *Crassostrea gigas* (Duperthuy et al., 2011). Thus, the factors that mediate the intracellular survival of *V. cholerae* in protozoa and their link with the pathogenic lifestyle of this bacterium are being revealed.

## Intra-phagosomal Factors in Protozoa and the Innate Immune Defense of the Gastrointestinal Tract: Similar Stressors Encountered in Both Environments

**Figure 1:**
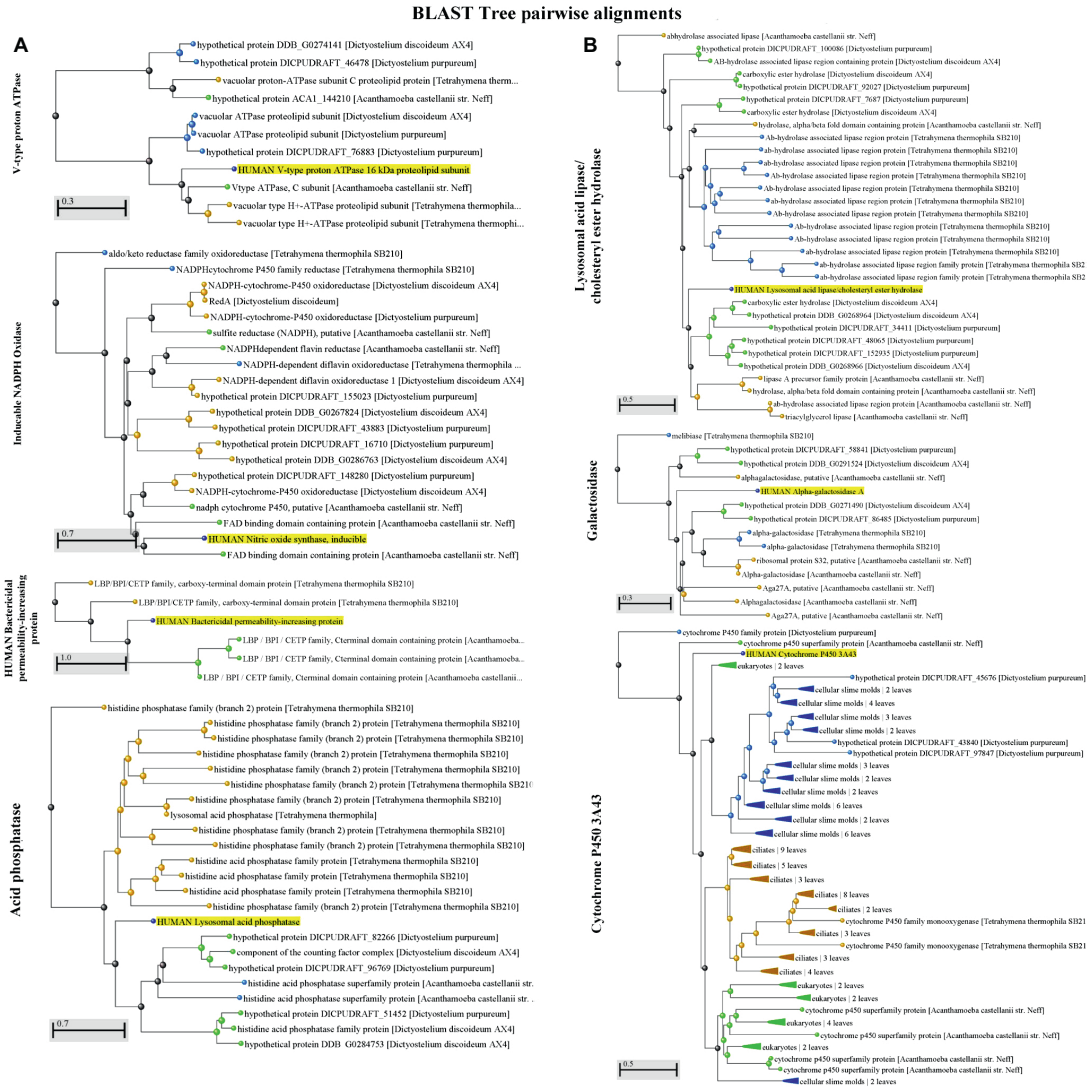
**(A)** A Blast Tree View of potential factors [V-type proton ATPase, inducible NADPH oxidase, and the human bactericidal permeability-increasing protein (BPI)] encountered in both protozoa and humans (highlighted) that contribute to the killing of *Vibrio* spp. shows pairwise alignment between human proteins and those found in protozoa. Produced by NCBI Tree Viewer. **(B)** A Blast Tree View of potential factors [acid phosphatase (lysosomal acid lipase/ cholesteryl ester hydrolase), galactosidase, cytochrome P450 3A43, and acid phosphatase] encountered in both protozoa and humans (highlighted) that contribute to the killing of *Vibrio* spp. shows pairwise alignment between human proteins and those found in protozoa. Produced by NCBI Tree Viewer.

Phagosomes of bacterivorous protozoa use mechanisms of killing and digestion of bacteria that are highly conserved in eukaryotic cells. Intracellular digestion begins with a reduction in the pH in order to create an acidic environment required for the proper activity of various antibacterial compounds that are vital for the digestion of bacteria. Many of these compounds have been described: (1) AMPs, amphipathic peptides that disrupt the integrity of the cell membrane, (2) ROS/RNS, also involved in the loss of membrane integrity as well as DNA damage in bacteria, and (3) proteolytic enzymes such as endopeptidases and lipases, required for the digestion of macromolecules (Flannagan et al., 2009). Here we will describe how the factors used by the innate immune system of the GI tract: the acidic environment of the stomach, ROS/RNS compounds produced after the breakdown of macromolecules in the presence of low pH and bile, proteolytic enzymes such as proteases, peptidases, lipases, amylases, and nucleases, and AMPs that are synthesized by the GI epithelium are also encountered inside phagocytic cells (Figure 1). It is likely that key factors used by *Vibrio* spp. to resist the intracellular environment in protozoa might also serve to protect cells against stressors in the GI tract. Here the mechanisms displayed by *Vibrio* spp. to resist such stressors and how this can affect the infective cycle of the model pathogen *V. cholerae* will be explored.

### Low pH

The acidification of phagosomes containing bacteria is a critical step for intracellular digestion in phagocytic cells. Under normal conditions, early phagosomes become acidified by the action of specific proteins located on the surface of phagosomes. The vacuolar V-ATPase is a highly conserved enzyme that transports H^+^ ions (Forgac, 1999; Toei et al., 2010) and is present in the phagosomes of protozoa and also on the surface of human GI cells (Figure 1). At the late stages of phagosome maturation, the low pH of the phagosome enables the fusion with the lysosome, an acidic organelle that contains enzymes that are crucial for the complete digestion of bacteria and macromolecules. Similarly, part of the initial steps in the digestion of macromolecules as well as the inactivation of pathogenic microorganisms in mammals takes place in the stomach, an environment that is characterized by a low pH. Here, the exposure to the acidic environment (due to hydrochloric acid) and the production of ROS/RNS in the gastric environment causes loss of membrane integrity and DNA damage in bacteria (Conner et al., 2016). Together, these facts highlight that acidification is an important conserved strategy used by different organisms to inactivate and digest bacteria.

*V. cholerae* expresses several survival strategies to adapt to acidic and oxidative conditions. Aggregation (or suspended biofilms) and biofilm formation have been reported to physically protect *V. cholerae* from acid stress (Zhu and Mekalanos, 2003) due to the strong protection given by the biofilm matrix that protects *V. cholerae* from various stressors, including antibiotics and ROS (Mankere et al., 2018; Wang et al., 2018). Another mechanism providing resistance to low pH is the activation of the acid tolerance response (ATR). In *V. cholerae*, the ATR is controlled by the modulation of the *cadBA* operon that is activated by the ToxR-like protein CadC (Merrell and Camilli, 2000). *cadA* is an infection-induced gene in *V. cholerae* that encodes a lysine decarboxylase (CadA) required for the active efflux of H^+^ ions from the bacterial cytoplasm to the extracellular space (Merrell and Camilli, 1999). The lysine cadaverine/antiporter (CadB) works together with CadA in the presence of high concentrations of H^+^ to provide resistance to acidic environments (Merrell and Camilli, 2000). Under acidic conditions, CadB catalyzes the uptake of lysine, which in combination with H^+^ ions forms cadaverine in the cell cytoplasm, a polyamine that is excreted outside of the cell by the same antiporter. In addition, the activation of ATR in *V. cholerae* can be mediated by ToxR in the presence of organic acids. It was shown that the ectopic expression of the ToxR-regulated outer membrane OmpU is sufficient to overcome the reduction in ATR that occurs in a ∆*toxR* mutation (Merrell et al., 2001).

Reports have shown that the adaptation of *V. cholerae* to low pH before infection causes a significant induction of the ATR system resulting in improved intestinal colonization (Merrell et al., 2002). However, it was shown that this colonization advantage is not due to increased survival of *V. cholerae* to the stomach environment or to the expression of colonization or virulence factors (Angelichio et al., 2004). It is believed that acid-adapted *V. cholerae* have a growth advantage over non-adapted cells and that this growth advantage is responsible of the hyperinfective phenotype *in vivo*, since the fitness advantage of acid-adapted *V. cholerae* could not be confirmed *in vitro* (Angelichio et al., 2004). Interestingly, it was recently shown that *V. cholerae* shows an increased resistance to low pH when contained in EFVs released by ciliated protozoa and also displays an *in vitro* growth advantage in high nutrient and temperature conditions and *in vivo* colonization advantage in the infant mouse colonization model (Espinoza-Vergara et al., 2019).

### Reactive Oxygen and Nitrogen Species

Under normal physiological conditions, the human body produces small amounts of ROS/RNS in the GI tract due to chemical reactions between oxygen and nitrogen components in the presence of acids or bile (Davies et al., 2011; Aviello and Knaus, 2018). ROS/RNS are oxidative species that can directly damage the DNA of microorganisms, thereby acting as a natural antimicrobial barrier. Human professional phagocytes and amoeba are known to produce ROS/RNS such as nitric oxide (NO) and hydrogen peroxide (H_2_O_2_) inside phagosomes as an antibacterial strategy (Zhang and Soldati, 2013; Di Meo et al., 2016). Indeed, the production of ROS in the phagosome of the amoeba *Dictyostelium discoideum* has been visualized and quantified (Zhang and Soldati, 2013). Furthermore, it is known that the intestinal epithelial layer also produces NO by the induction of oxide synthases (iNOS) (Eckmann et al., 2000). To resist RNS/ROS, pathogenic bacteria such as *V. cholerae* display specific factors such as *hmpA* and *nnrS*, two genes under the control of the σ^54^-dependent transcriptional regulator NorR (Stern et al., 2012). Deletion of either *hmpA* or *nnrS* causes a significant reduction in long-term colonization of *V. cholerae* in the adult mouse model, showing that RNS is an important barrier to *V. cholerae* infection *in vivo* (Stern et al., 2012). Another strategy to resist ROS used by many microorganisms as well as eukaryotic cells is the expression of catalases, superoxide dismutase (SOD), and alkyl superoxide reductase subunit C’s (Imlay, 2008). These enzymes break down ROS into non-damaging sub-products such as H_2_ and O_2_. In *V. cholerae*, OxyR, and two catalases KatG and KatB are involved in the resistance to ROS (Wang et al., 2013). Thus, the factors used by *V. cholerae* to resist RNS/ROS may facilitate the survival of this bacterium inside phagotrophic protozoa as well as within the human intestinal tract.

### Antimicrobial Peptides

Another cause of mortality for pathogenic bacteria in the host is the presence of host-derived AMPs. Production of these molecules can be mediated in the human host by several phagocytes and epithelial cells (Diamond et al., 2009), e.g., macrophages and the GI epithelium. In mammals, the two main classes of AMPs are defensins and cathelicidins (Dorin et al., 2015). Most of the AMPs act by disrupting and permeabilizing the cell membrane, causing loss of viability. Some examples of AMPs produced in the human intestinal tract are: α-defensins: human neutrophil peptides 1-4 (HNP1-4), and human defensin 5 and 6 (HD5 and HD6); β-defensins: beta defensin 1 to 4 (hBD1-4); cathelicidin: LL-37/h-CAP18 (human cathelicidin antimicrobial peptide 18 kDa); other AMPs: bactericidal/permeability-increasing protein (BPI), chemokines CCL14, CCL15, and CCL20/macrophage-immflamatory-protein-3α (Muniz et al., 2012). Importantly, AMPs are also produced by phagocytic cells in order to arrest the growth and inactivate bacteria. As shown in Figure 1, heterotrophic protozoa such as *Tetrahymena* spp., *Dictyostelium* spp., and *Acanthamoeba* spp. encode proteins with high similarities to BPI, an important bactericidal and LPS neutralizing AMP released by human neutrophils (Calafat et al., 2000) and GI epithelial cells (Canny and Colgan, 2005).

Polymyxin B as well as other cationic AMPs (CAMPs) has been widely used to screen for AMP resistance in *V. cholerae* and other Gram-negative bacteria. Genes related to the modification of the lipid A portion of LPS (Henderson et al., 2014), the outer membrane porin B, and OmpT (Mathur and Waldor, 2004) as well as the VexAB system (Bina et al., 2006) are important for *V. cholerae* resistance to CAMPs. Lipid A acylation has been reported to play an important role in the resistance to CAMPs in bacterial pathogens such as *V. cholerae*, *Salmonella enterica*, *Escherichia coli*, and *Helicobacter pylori* (Guo et al., 1998; Band and Weiss, 2014). In *V. cholerae*, mutation in the acyltransferase gene, *msbB,* resulted in a significant reduction in the resistance to polymyxin B and impairment in colonization of the small intestinal tract of the infant mouse (Matson et al., 2010), indicating that CAMPs are an important line of defense against *V. cholerae*. In addition, genes involved in the aminoacylation of lipid A encoded within the *almEFG* operon, are essential for resistance to CAMPs (Henderson et al., 2014). Recently, AlmG, a glycosyltransferase, positively regulated by the response regulator, CarR (Bilecen et al., 2015), has been identified to be responsible for polymyxin B resistance in pandemic *V. cholerae* (Henderson et al., 2017). It is known that a reduction in the aminoacylation/phosphorylation of lipid A results in a decrease in the negative charge surface of the bacterial outer membrane, causing an increased affinity for CAMPs with target molecules (Steimle et al., 2016).

Similar to the modification of lipid A, the expression of major outer membrane proteins in *V. cholerae* is critical for resistance to CAMPs. OmpU, the major outer membrane protein of *V. cholerae*, plays a key role in the resistance to polymyxin B and other CAMPs such as P2, an active peptide derived from BPI (Mathur and Waldor, 2004). It has been proposed that the interaction between OmpU and AMPs leads to the activation of the stress response mediated by the sigma factor σ^E^, resulting in increased survival (Mathur et al., 2007). In general terms, stress responses in bacteria lead to the activation of specific pathways in response to different stressors such as starvation, biocides, and temperature in order to maintain cell viability. Another mechanism for resistance to AMPs is the activation of the VexAB system. As described previously, VexAB is an efflux system in *V. cholerae* involved in the resistance to antibiotics, such as polymyxin B and also tensoactive molecules such as SDS and Triton-X 100 (Bina et al., 2006). Interestingly, deletion of ∆*vexAB* reduced CT production, expression of virulence factors, and colonization (Bina et al., 2008), suggesting that this systems like the VexAB might be important to *V. cholerae* survival in the presence of AMPs inside phagosomes/food vacuoles in protozoa and also in the human intestinal tract.

### Digestive and Other Enzymes

Lysosomal acid lipase (gastric lipase in the stomach), acid phosphatase and galactosidase are three digestive enzymes present in both protozoa and the GI tract (Figure 1). Although there is a lack of information about the impact of these enzymes on the pathogenicity of bacteria, the bacterial resistance to these factors in their primary aquatic habitat might promote pathogen’s growth in the intestinal tract. This idea is supported by the fact that the maintenance of normal levels of digestive enzymes such as alkaline phosphatase in the gut contributes to the growth of beneficial commensal bacteria and prevents the growth of pathogenic microorganisms (Malo et al., 2010).

Interestingly, the presence of cytochrome P450, an enzyme involved in the production of steroid hormones, cholesterol, fatty acids, and bile acids in humans is also present in protozoa (Zimniak and Waxman, 1993). It is known that the presence of bile acids induces the expression of virulence factors such as the cholera toxin in *V. cholerae* (Hung and Mekalanos, 2005). Despite the fact that the biosynthesis of cholesterol (bile acids precursor) has not been reported in protozoa such as *Tetrahymena* spp., similar organic acids potentially produced by protist hosts might induce the expression of virulence factors in pathogenic *Vibrio* spp. and other bacteria.

The resistance of *Vibrio* spp. to the factors encountered inside of the phagosomes/food vacuoles in heterotrophic protozoa might serve as a pre-adaptation niche before entering a host. In addition to the physical protection that a protozoa might confer to intracellular pathogens, as has been previously suggested, the passage of bacteria within protozoa might activate specific factors used to resist the strategies that also contribute to the inactivation of bacteria within mammalian hosts. Thus, the adaptation and resistance to the intracellular environment in protozoa may positively impact on the infective cycle of pathogenic *Vibrio* spp., possibly by increasing the number of viable cells that reach the site of infection or by enhanced pathogenicity.

## The Impact of Protozoan Predation on Virulence

The interaction of bacteria with protozoa has been correlated with increased pathogenicity, and thus, protists hosts have been suggested to be “Trojan horses” protecting and disseminating pathogens in the environment (Barker and Brown, 1994; Denoncourt et al., 2014). For example, Gram-negative pathogens such as *S. enterica*, *E. coli*, and *Listeria monocytogenes* can survive and remain inside *A. castellanii* cysts where they are more tolerant to antibiotics and low pH (Lambrecht et al., 2015). Similar protection was shown for *Legionella pneumophila,* with increased resistance to chlorine when inside of *A. polyphaga* cysts (Kilvington and Price, 1990). It is known that eukaryotic membranes might act as a physical barrier to biocides, a fact that might explain this effect. In contrast, bacterial adaptation to the intracellular environment can also result in increased pathogenicity through improved resistance to antimicrobials and induction of virulence factors. For example, the use of divalent metals such as copper and zinc is a conserved antimicrobial mechanism against bacteria in both amoeba and macrophages (German et al., 2013). Thus, resistance to copper and/or zinc might lead to increased bacterial virulence *in vivo*. In *E. coli* and *Pseudomonas aeruginosa*, genes encoding copper resistance are related to grazing resistance against *D. discoideum* (Hao et al., 2016). In *Campylobacter jejuni*, the efflux system CmeABC that confers resistance to antibiotics, might also be involved in metal detoxification and increased virulence (Vieira et al., 2017).

**Figure 2:**
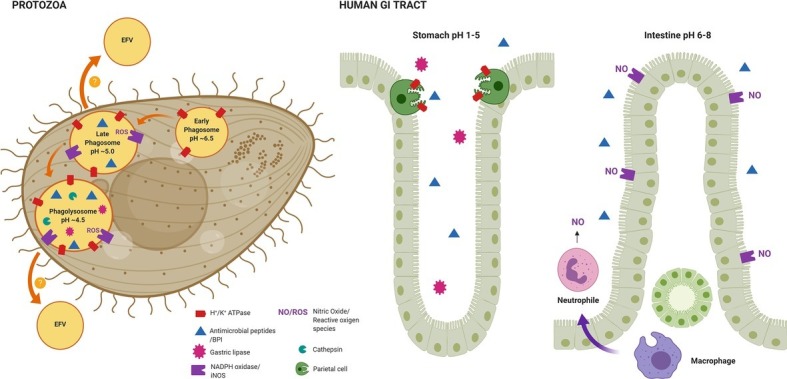
Representation of the conserved factors required for the inactivation and digestion of bacteria used by both protozoa and the innate defense system of the human GI tract. The maturation of bacterial-containing phagosomes in *Tetrahymena* (Protozoa), a process that depends on acidification. As shown, different factors are recruited at different stages of the phagosome maturation process. Some pathogenic bacteria, such as *Vibrio* spp., are able to resist the digestion process and are expelled in EFVs to the extracellular environment, a condition where they show a hyperinfective phenotype *in vivo*. From left to right in human GI tract, the antimicrobial strategies deployed by the stomach and the intestinal tract. The factors represented here are highly similar to the ones encountered in phagocytic protozoa, thus, the pre-adaptation to such stressors within protozoa might be crucial for pathogenic bacteria to survive and multiply within the human GI tract. The figure was created with BioRender.com.

The transit of bacteria through protozoa has been linked to increased hyperinfectivity in pathogenic bacteria. For example, *S. enterica* and *L. pneumophila* recovered after exposure to *A. castellanii* display hyperinvasive phenotypes during *in vivo* infection (Cirillo et al., 1994; Rasmussen et al., 2005). Similarly, mice infections performed with *Mycobacterium ulcerans* previously co-incubated with *A. polyphaga* led to enhanced pathogenicity (Azumah et al., 2017). In the case of *Vibrio* spp., it has recently been shown that their release in EFVs to the extracellular environment results in bacterial growth and colonization advantage *in vitro* and *in vivo*, respectively (Espinoza-Vergara et al., 2019). In addition, the use of a critical virulence factor in *V. cholerae*, OmpU, involved in colonization and resistance to low pH, AMPs, and bile, was shown to be involved in the release of EFVs from protozoa. This fact illustrates how a factor involved in the resistance to stressors encountered in the protozoan phagosome and within the human host enhances the survival and potentially increases the infectivity of *V. cholerae* (Figure 2). Thus, the release of *Vibrio* spp. in EFVs as well as the intracellular adaptation to the presence of biocides and induction of virulence in bacteria can lead to fitness advantages during infection of a host.

## Conclusions and Future Perspective

Taken together, this review highlights that the strategies used to digest and inactivate bacteria in both protozoa and the GI tract of the human host are highly conserved and further emphasize how the resistance to the intracellular digestion in protozoa might enhance the pathogenicity of *Vibrio* spp. More research regarding the impact on the infection cycle of intracellular exposed *Vibrio* spp. is fundamental to further understanding the mechanisms that result in hyperinfectivity. This will not only enable us to identify key environmental clues that enable the pathogenicity of important pathogens such as *V. cholerae* but also will contribute to understanding whether the activation of hyperinfectivity is a conserved response in other pathogenic bacteria that interact with protozoa.

The packaging of multiple bacteria into phagosomes by protozoa might explain how pathogenic *Vibrio* spp. have acquired specific factors that increase the fitness during infection. In the past, increased conjugation rates have been shown for *E. coli* contained in *Tetrahymena* phagosomes (Schlimme et al., 1997; Matsuo et al., 2010). Thus, horizontal gene transfer in protozoa might be a crucial step for pathogenic bacteria to increase fitness in both the environment and during infection of a host. As next-generation sequencing continues to become more affordable, the evaluation of horizontal gene transfer of pathogenic *Vibrio* spp. in protozoa becomes possible, thus adding another layer of potential selection pressure on the development of virulence.

## Author Contributions

All authors listed have made a substantial, direct and intellectual contribution to the work, and approved it for publication.

### Conflict of Interest

The authors declare that the research was conducted in the absence of any commercial or financial relationships that could be construed as a potential conflict of interest.
